# Genetic Scale for Predicting the No-Reflow Phenomenon in Myocardial Infarction

**DOI:** 10.17691/stm2025.17.5.05

**Published:** 2025-10-31

**Authors:** I.G. Pochinka, A.A. Frolov, K.V. Kuzmichev, N.A. Shchelchkova, V.I. Pershin, N.S. Maximova, M.L. Budkina, I.V. Predeina, I.A. Frolov, M.G. Kashtanov

**Affiliations:** MD, DSc, Associate Professor, Head of the Department of Endocrinology and Internal Medicine; Privolzhsky Research Medical University, 10/1 Minin and Pozharsky Square, Nizhny Novgorod, 603005, Russia; MD, PhD, Associate Professor, B.A. Korolev Department of Hospital Surgery; Privolzhsky Research Medical University, 10/1 Minin and Pozharsky Square, Nizhny Novgorod, 603005, Russia; Assistant, Department of Endocrinology and Internal Medicine; Privolzhsky Research Medical University, 10/1 Minin and Pozharsky Square, Nizhny Novgorod, 603005, Russia; PhD, Head of the Central Research Laboratory; Privolzhsky Research Medical University, 10/1 Minin and Pozharsky Square, Nizhny Novgorod, 603005, Russia; Junior Researcher, Molecular Genetics Department, Central Research Laboratory; Privolzhsky Research Medical University, 10/1 Minin and Pozharsky Square, Nizhny Novgorod, 603005, Russia; Junior Researcher, Molecular Genetics Department, Central Research Laboratory; Privolzhsky Research Medical University, 10/1 Minin and Pozharsky Square, Nizhny Novgorod, 603005, Russia; MD, PhD, Associate Professor, Department of Endocrinology and Internal Medicine; Privolzhsky Research Medical University, 10/1 Minin and Pozharsky Square, Nizhny Novgorod, 603005, Russia; Biologist, Genetic Diagnostics Laboratory, Center for Agrobiotechnology, Institute of Fundamental Medicine; Privolzhsky Research Medical University, 10/1 Minin and Pozharsky Square, Nizhny Novgorod, 603005, Russia; Physician, Department of X-ray Surgical Methods of Diagnosis and Treatment; City Clinical Hospital No.13 of the Avtozavodsky District of Nizhny Novgorod, 51 Patriotov St., Nizhny Novgorod, 603018, Russia; MD, PhD, Physician, Department of X-ray Surgical Methods of Diagnosis and Treatment; Regional Clinical Hospital No.1, 55 Kotovskogo St., Tyumen, 625023, Russia; Researcher, Laboratory of X-ray Surgical Methods of Diagnosis and Treatment; Ural Federal University named after the First President of Russia B.N. Yeltsin, 19 Mira St., Ekaterinburg, 620002, Russia

**Keywords:** myocardial infarction, no-reflow phenomenon, percutaneous coronary intervention, single nucleotide polymorphism

## Abstract

**The aim of the study is** to investigate the association of the selected single nucleotide polymorphisms (SNPs) with the development of the no-reflow phenomenon during percutaneous coronary intervention (PCI) in patients with ST-segment elevation myocardial infarction (STEMI) and to create a genetic scale for predicting this complication.

**Materials and Methods:**

A single-center matched case–control study was conducted. The study included 80 STEMI patients: 40 (50%) with no-reflow and 40 (50%) without no-reflow (1:1 matching by sex and age). No-reflow was defined as TIMI flow grade <3 or Myocardial blush grade <2 after PCI. The following SNPs were assessed: rs4961 (*ADD1*), rs699 and rs4762 (*AGT*), rs5186 (*AGTR1*), rs1403543 (*AGTR2*), rs1799998 (*CYP11B2)*, rs5443 (*GNB3*), rs2070744 and rs1799983 (*eNOS*), rs5370 (*EDN1*), rs1799963 (*F2*), rs6025 (*F5*), rs6046 (*F7*), rs5985 (F13), rs1800790 (*FGB*), rs1126643 (*ITGA2*), rs5918 (*ITGB3*), rs1799762 (*PAI-1*), rs1801133 and rs1801131 (*MTHFR*), rs1805087 (*MTR*), and rs1801394 (*MTRR*).

**Results:**

The following SNPs were associated with the development of the no-reflow phenomenon: rs4961 (genotype GT or TT) in the *ADD1* gene, rs1799998 (CC) in the *CYP11B2* gene, and rs1801133 (CC) in the *MTHFR* gene (p<0.05, McNemar’s test). These SNPs were combined into a genetic prognostic scale, where 1 point was assigned for each genotype associated with no-reflow. The positive predictive value for the maximum score (3 points) was 0.91. The area under the ROC curve was 0.724 (0.611–0.838). The odds ratio for no-reflow development was 5.39 (1.09–26.66) per point (p=0.04; multivariate analysis using conditional logistic regression).

## Introduction

Despite advances in modern pharmacotherapy for cardiovascular disease prevention, the incidence of myocardial infarction (MI) remains consistently high. The most effective treatment method is percutaneous coronary intervention (PCI); its widespread implementation has radically reduced MI mortality and the frequency of its complications [1].

The no-reflow phenomenon is observed in approximately 15% of patients with ST-segment elevation MI (STEMI) during PCI. The no-reflow phenomenon is defined as a condition when the restoration of the lumen of the epicardial infarct-related artery (IRA) during PCI does not lead to adequate myocardial perfusion due to the presence of coronary microvascular obstruction. The development of this complication significantly increases the risk of death and progression of chronic heart failure [2].

It is known that several pathological mechanisms lead to the formation of the no-reflow phenomenon. The most common causes are the following: initially severe ischemic injury, distal microembolization by thrombus or atherosclerotic plaque components, and endothelial dysfunction [2, 3]. The diversity of pathophysiological mechanisms underlying the no-reflow phenomenon causes significant difficulties in the prevention and effective treatment of this complication.

To improve the effectiveness of no-reflow prevention, several prognostic scales have been created that consider various clinical, angiographic, and laboratory predictors. The most well-known models are following: the scale by Wang et al. [4]; the No-reflow score [5]; the model by Xiao et al. [6]; the model by Bessonov et al. [7]; the RECOVER score [8]; and the PIANO score [9]. However, it should be emphasized that none of these scales have external validation on independent data. Besides, the prediction accuracy of the mentioned models remains moderate, with an average area under the ROC curve (AUC) being about 0.800. It is probably explained by the fact that the proposed scales do not include parameters reflecting the individual patient’s predisposition to no-reflow development and do not assess all known pathogenetic mechanisms. The majority of these models include factors related to the volume of coronary thrombotic mass or total ischemic time, but none of them account for the presence of endothelial dysfunction or platelet state. We hypothesize that personal genetic characteristics may influence the risk of developing the no-reflow phenomenon. Accordingly, prognostic models that consider specific genetic determinants of no-reflow may have greater predictive accuracy.

A small number of studies describe the association of certain single nucleotide polymorphism (SNP) variants with the state of the coronary microvasculature. In a large study by Yoshino et al. [10], an association of certain SNPs with coronary microcirculation dysfunction is described in stable patients with angina symptoms but without significant obstruction of the epicardial coronary arteries. In this study, such an association was found for SNP rs3025039 in the *VEGFA* gene and SNPs rs10757274, rs2383206, rs1004638, rs2383207, rs1333049 in the *CDKN2B-AS1* gene. The odds ratio (OR) for the presence of microvascular dysfunction for these SNPs ranged from 1.44 to 1.68.

Some studies have investigated the association of certain SNPs with the development of no-reflow in MI patients. For example, in the study by Dharma et al. [11], the presence of the AA genotype in SNP rs2305619 of the *PTX3* gene increased the chance of no-reflow development by 4.48 times. Fracassi et al. [12] associated the TT genotype of SNP rs1333040 in the *CDKN2B-AS1* gene with no-reflow development. However, given the multifactorial no-reflow pathogenesis, it is reasonable to assume that accurate prediction requires the simultaneous consideration of several genetic factors associated with different noreflow mechanisms. In other words, a genetic scale for no-reflow prediction should be created.

SNPs associated with no-reflow mechanisms that cannot be assessed based on parameters routinely available in clinical practice are of the greatest interest for inclusion in a potential genetic scale. In our work, we decided to focus precisely on such genetic determinants (for details, see the “Interpretation of results” subsection) [2, 3, 10–14].

**The aim of the study** is to investigate the association between the selected SNPs of the following genes: of the renin-angiotensin-aldosterone system, endothelial function, folate cycle, platelet function, and hemostasis system, with the no-reflow development during PCI in STEMI patients, as well as to create a genetic scale for predicting this complication.

## Materials and Methods

A single-center case–control study was conducted.

The study was approved by the local Ethics Committee of Privolzhsky Research Medical University (Protocol No.5 dated April 8, 2022). The study protocol was registered at clinicaltrial.gov (NCT05355532). The study was conducted in accordance with the standards of Good Clinical Practice and the principles of the Helsinki Declaration (2024). All participants signed a voluntary informed consent form.

### Study participants and data sources

Patient enrollment was conducted during 2022–2023. 80 patients with type I STEMI who had undergone emergency PCI were selected: 40 (50%) patients were in the no-reflow group (case) and 40 (50%) were in the group without no-reflow (control). The groups were matched by sex and age (±5 years) in a 1:1 ratio and were formed with the use of the “matched pairs” method. All sequentially admitted patients who met the inclusion criteria and had no exclusion criteria were included in the study.

The exclusion criteria were the following: subacute MI (more than 48 h from the onset of anginal status) or early post-infarction angina; dissection, perforation, or acute intraoperative thrombosis of the IRA; MI related to a revascularization procedure (type IV); death during PCI not caused by no-reflow development; concomitant terminal pathology unrelated to the underlying disease with an expected life expectancy of less than 1 month; initial limitation of myocardial perfusion due to the presence of cardiogenic shock that developed before PCI.

The no-reflow development in the IRA was confirmed by angiographic criteria at the end of PCI: TIMI flow grade [15] less than 3 or Myocardial blush grade [16] less than 2.

Signing a voluntary informed consent form and patient inclusion in the study took place in the catheterization laboratory after PCI completion. All data analyzed in the study were collected prospectively.

### Genetic analysis

As potential risk factors for noreflow development, there were selected 5 groups of SNPs associated with endothelial function genes, the renin-angiotensin-aldosterone system, the coagulation cascade, platelet function, and folate metabolism (22 SNPs in total). The selection was based on literature data indicating that these SNPs were associated with biochemical mechanisms being the components of noreflow pathogenesis (for details, see the “Interpretation of results” subsection) [2, 3, 10–14]. The list of analyzed SNPs in the format “gene group: SNP identifier (gene, encoded protein)” is shown below.

Endothelial function genes are the following: rs4961 (*ADD1*, α-adducin), rs5443 (*GNB3*, G-protein β-3 subunit), rs2070744 (*eNOS*, endothelial NO synthase), rs1799983 (*eNOS*, endothelial NO synthase), and rs5370 (*EDN1*, endothelin-1). Renin-angiotensin- aldosterone system genes included the following ones: rs4762 (*AGT*, angiotensinogen), rs699 (*AGT*, angiotensinogen), rs5186 (*AGTR1*, angiotensin II type 1 receptor), rs1403543 (*AGTR2*, angiotensin II type 2 receptor), and rs1799998 (*CYP11B2*, aldosterone synthase). Coagulation cascade genes are the following: rs1799963 (*F2*, prothrombin), rs6025 (*F5*, proaccelerin), rs6046 (*F7*, proconvertin), rs5985 (*F13*, fibrinase), and rs1800790 (*FGB*, fibrinogen). Platelet function genes include the following ones: rs1126643 (*ITGA2*, α-2-integrin), rs5918 (*ITGB3*, β-3-integrin), and rs1799762 (*PAI-1*, serpin). Folate metabolism genes are the following: rs1801133 (*MTHFR*, methylenetetrahydrofolate reductase), rs1801131 (*MTHFR*, methylenetetrahydrofolate reductase), rs1805087 (*MTR*, B12-dependent methionine synthase), and rs1801394 (*MTRR*, methionine synthase reductase).

For genetic analysis, peripheral blood was collected. Ethylenediaminetetraacetate salts were used as an anticoagulant at a final concentration of 2.0 mg/ml. Samples were stored at 2–8°C and transported to the laboratory within 24 h.

Genetic testing was performed by real-time polymerase chain reaction with high-resolution melting curve analysis using TaqMan fluorescent probes and the “hot start” amplifier function. The following reagent kits were used: CardioGenetics Hypertension, CardioGenetics Thrombophilia, and Genetics of Folate Metabolism (all produced by DNA-Technology, Russia), as well as the SNP-Express-Cardiogenetics kit for detecting the Lys198Asn SNP in the *EDN1* gene (Lytech, Russia). To exclude genotyping errors, all studied SNPs for all patients included in the study were re-genotyped by two independent geneticists.

As all analyzed SNPs were located in autosomes (paired chromosomes), the allelic variants (combinations) of these SNPs were determined during the genetic analysis. A conclusion was made about the presence of reference (“wild”, more common) alleles, alternative (“mutant”, less common) alleles, or their combination. Considering the study design (case–control), binary genetic models were used in the subsequent group comparison: a recessive model (groups are compared by the proportion of patients having at least one alternative allele in the analyzed SNP) and a dominant model (groups are compared by the proportion of patients having at least one reference allele in the analyzed SNP). All patients included in the study resided in the European part of Russia. Information on which allele was the reference for these patients was taken from the international SNP database — dbSNP (hosted by the National Center for Biotechnology Information (NCBI), USA).

### Indicators and outcomes

Treatment of all patients was carried out in accordance with current clinical guidelines [1]. In addition to matching patients by sex and age, to control confounders and to prevent “bias” in the study results, indicators being the no-reflow development predictors and included in the known prognostic scales [4–9] were considered in the statistical analysis. A number of the parameters used in these scales were not analyzed for organizational reasons (see the “Limitations and prospects” section for more details).

In addition to the previously mentioned TIMI flow grade [15] and Myocardial blush grade [16], the study utilized the Rentrop scale [17] to assess the severity of collateral arteries to the IRA, the Killip classification [18] to determine the severity of acute heart failure, and the Thrombus burden classification [19] to measure the severity of IRA thrombosis.

The observation period corresponded to the duration of hospitalization. The development of Q-wave MI and mortality were recorded. On the 10^th^ day of hospitalization, echocardiography was performed with left ventricular ejection fraction measurement using the Simpson method.

### Statistical methods

The required sample size (80 patients) was calculated based on the following parameters: alpha error rate of 5%, study power of 80%, patient ratio in comparison groups of 1:1, minimum OR for detection of 4.0; prevalence range of the planned SNP variants in the population from 12% to 69% (mean 50%). Of the 40 pairs planned for recruitment, 20 pairs should be discordant (patients in a pair should differ in the presence or absence of the predictor).

In statistical analysis, the Lilliefors test was used to determine the distribution pattern. For group comparisons in univariate analysis, the McNemar and Wilcoxon tests were used. For multivariate analysis and confounder control, conditional logistic regression or a fixed-effects model were used. To assess the conformity of allele distributions with the Hardy–Weinberg law, the Pearson chi-square test was used. Differences were considered statistically significant at p<0.05. Quantitative data were presented as medians and interquartile ranges (Me [Q1; Q3]); qualitative data were presented as absolute values and percentages (n (%)).

The laboratory and instrumental data contained missing values, classified as “missing at random” (MAR). To handle these missing values, multivariate imputation by chained equations (MICE) with classification and regression trees (CART) was used [20]. The genetic analysis results for a small number of patients also contained missing data. The cause of missing data was hemolysis of the blood sample during transportation, which was classified as “missing completely at random” (MCAR). Patients with missing genetic data were excluded from the corresponding analyses.

Statistical analysis was performed using the RStudio programming environment (Posit Software, USA, version 2023.06.1+524). The following libraries were used: DescTools, dlookr, dplyr, exact2x2, flextable, ggplot2, gtsummary, HardyWeinberg, MESS, mice, reporter, reshape2, ROCit, sjPlot, stringr, survival, and tibble.

## Results

### Clinical characteristics of the patients

The median age of the 80 patients included in the study was 65 [60; 72] years. 58 men (73%) and 22 women (27%) were included. The median length of hospitalization was 11 [8; 13] days. 8 patients (10%) died during hospitalization; 6 of these patients died from progressive acute left ventricular failure, one from a mechanical complication of MI, and one from ventricular fibrillation.

In the sample of 80 patients selected for the study, missing data were noted in laboratory and instrumental parameters: neutrophils (1.3% of values were missing), glucose (2.5%), and left ventricular ejection fraction (32.5%).

Missing data were noted among the following SNPs analyzed: *AGT* (rs4762) — 1 (1%), *AGTR2* (rs1403543) — 1 (1%), *CYP11B2* (rs1799998) — 1 (1%), *eNOS* (rs2070744) — 1 (1%), *F7* (rs6046) — 2 (3%), *MTHFR* (rs1801133) — 2 (3%), *MTRR* (rs1801394) — 3 (4%), *AGT* (rs699) — 4 (5%), *AGTR1* (rs5186) — 4 (5%), *EDN1* (rs5370) — 4 (5%), *F2* (rs1799963) — 4 (5%), *F5* (rs6025) — 4 (5%), *F13* (rs5985) — 4 (5%), *ITGB3* (rs5918) — 4 (5%), *MTHFR* (rs1801131) — 4 (5%), *FGB* (rs1800790) — 7 (9%), *ITGA2* (rs1126643) — 7 (9%), *PAI-1* (rs1799762) — 7 (9%), *MTR* (rs1805087) — 7 (9%) missing values.

General characteristics of patients and comparison of study groups by no-reflow predictors and outcomes are presented in Table 1. Univariate analysis of group differences by the proportion of patients with different allelic variants of the studied SNPs is presented in Table 2.

**T a b l e 1:** General characteristics and comparison of groups by no-reflow predictors and outcomes

Indicator	Total sample (n=80)	No-reflow phenomenon		
		Absent (n=40)	Present (n=40)	^p^
***No-reflow predictors***				
Age (years)	65 [60; 72]	65 [61; 71]	66 [60; 73]	0.69
Sex:				
female	22(27)	11(27)	11(27)	1.00
male	58(73)	29(73)	29(73)	
History of coronary heart disease	31(39)	14(35)	17(43)	0.66
Acute heart failure, Killip class:				
1	78(98)	40(100)	38 (95.0)	
2	1(1)	0	1 (2.5)	0.37
3	1(1)	0	1 (2.5)	
4	0	0	0	
Systemic thrombolytic therapy	9(11)	3(8)	6(15)	0.51
Total ischemic time (h)	6 [4; 12]	5 [4; 11]	7 [4; 13]	0.21
Infarct-related artery lesion in the LMCA/LAD	36(45)	18(45)	18(45)	1.00
Collaterals to IRA (grade):				
0	46(58)	16(40)	30(75)	
1	20(25)	14(35)	6(15)	**0.02**
2	14(18)	10(25)	4(10)	
IRA thrombosis (grade):				
0	3(4)	2 (5.0)	1 (2.5)	
1	15(19)	9 (22.5)	6 (15.0)	
2	8(10)	5 (12.5)	3 (7.5)	0.11
3	8(10)	5 (12.5)	3 (7.5)	
4	4(5)	1 (2.5)	3 (7.5)	
5	42(52)	18 (45.0)	24 (60.0)	
TIMI flow grade in IRA before PCI (grade):				
0	54(67)	25(62)	29 (72.5)	
1	3(4)	2(5)	1 (2.5)	0.24
2	16(20)	7(18)	9 (22.5)	
3	7(8)	6(15)	1 (2.5)	
IRA diameter (mm)	3.5 [3.0; 3.6]	3.0 [3.5; 3.6]	3.5 [3.5; 4.5]	**0.02**
Lesion length in IRA (mm)	30 [26; 53]	30 [23; 48]	36 [26; 55]	0.22
IRA pre-dilation	52(65)	30(75)	22(55)	0.10
Glucose on admission (mmol/L)	8.2 [6.8; 12.3]	8.1 [6.7; 9.5]	8.3 [6.8; 14.4]	0.13
Neutrophils on admission (10^9^ U/L)	7.8 [6.2; 9.7]	7.1 [6.1; 9.1]	8.1 [6.4; 10.8]	0.22
***Outcomes***				
Q-MI	74(93)	34(85)	40(100)	**0.03**
Left ventricular ejection fraction (%)	48 [41; 53]	50 [43; 53]	47 [38; 51]	0.23
In-hospital mortality	8(10)	2(5)	6(15)	0.26

N o t e. Qualitative data are presented as absolute values and percentages — n (%), quantitative data — as medians and interquartile ranges (Me [Q1; Q3]). MI — myocardial infarction; IRA — infarct-related artery; LMCA — left main coronary artery; LAD — left anterior descending artery; PCI — percutaneous coronary intervention; TIMI — thrombolysis in myocardial infarction (study group)

**T a b l e 2:** General characteristics and comparison of groups by proportion of patients with different allelic variants of single nucleotide polymorphisms

SNP identifier (gene, encoded protein)	Dominant genetic model					Recessive genetic model				
	Analyzed allelic variant	Total sample	No-reflow phenomenon			Analyzed allelic variant	Total sample	No-reflow phenomenon		
			Absent	Present	p			Absent	Present	p
***Endothelial function genes***										
rs4961 (*ADD1*, α-adducin)	GG or GT	67(84)	38(95)	29(73)	**0.03**	GT or TT	39(49)	14(35)	25(63)	**0.04**
rs5443 (*GNB3*, G protein β-3 subunit)	CC or CT	74(93)	36(90)	38(95)	0.68	CT or TT	49(61)	27(68)	22(55)	0.36
rs2070744 (*eNOS*, endothelial NO-synthase)	TT or TC	63(80)	32(82)	31(78)	0.77	TC or CC	51(65)	22(56)	29(73)	0.11
rs1799983 (*eNOS*, endothelial NO-synthase)	GG or GT	63(79)	33(83)	30(75)	0.58	GT or TT	42(53)	17(43)	25(63)	0.13
rs5370 (*EDN1*, endothelin-1)	GG or GT	73(96)	35(95)	38(97)	1.00	GT or TT	24(32)	10(27)	14(36)	0.45
***Renin-angiotensin-aldosterone system genes***										
rs4762 (*AGT*, angiotensinogen)	CC or CT	76(100)	37(100)	39(100)	—	CT or TT	44(56)	25(64)	19(48)	0.19
rs699 (*AGT*, angiotensinogen)	TT or TC	69(87)	31(79)	38(95)	0.11	TC or CC	32(42)	16(43)	16(41)	0.82
rs5186 (*AGTR1*, angiotensinogen II type 1 receptor)	AA or AC	68(89)	34(89)	34(89)	1.00	AC or CC	32(42)	19(50)	13(34)	0.21
rs1403543 (*AGTR2*, angiotensinogen II type 2 receptor)	GG or GA	49(62)	22(56)	27(68)	0.30	GA or AA	51(65)	24(62)	27(68)	0.79
rs1799998 (*CYP11B2*, aldosterone synthase)	CC or CT	63(80)	28(72)	35(88)	0.18	CT or TT	52(66)	32(82)	20(50)	**0.006**
***Coagulation cascade genes***										
rs1799963 (*F2*, prothrombin)	GG or GA	76(100)	40(100)	36(100)	—	GA or AA	3 (3.9)	3 (7.5)	0 (0)	—
rs6025 (*F5*, proaccelerin)	GG or GA	76(100)	40(100)	36(100)	—	GA or AA	6 (7.9)	2(5)	4(11)	0.69
rs6046 (*F7*, proconvertin)	TT or TA	73(94)	39(98)	34(89)	0.25	TA or AA	31(40)	19(48)	12(32)	0.38
rs5985 (*F13*, fibrinase)	GG or GT	68(89)	33(83)	35(97)	0.13	GT or TT	50(66)	29(73)	21(58)	0.48
rs1800790 (*FGB*, fibrinogen)	TT or TA	67(92)	33(87)	34(97)	0.37	TA or AA	33(45)	18(47)	15(43)	1.00
***Platelet function genes***										
rs1126643 (*ITGA2*, α-2-integrin)	CC or CT	61(84)	31(79)	30(88)	0.34	CT or TT	44(60)	21(54)	23(68)	0.27
rs5918 (*ITGB3*, β-3-integrin)	TT or TC	74(97)	39(98)	35(97)	1.00	TC or CC	37(49)	20(50)	17(47)	1.00
rs1799762 (*PAI*-1, serpin)	5G5G or 5G4G	50(68)	29(74)	21(62)	0.18	5G4G or 4G4G	62(85)	34(87)	28(82)	1.00
***Folate metabolism genes***										
rs1801133 (*MTHFR*, methylenetetrahydrofolate reductase)	CC or CT	72(92)	38(95)	34(89)	0.68	CT or TT	38(49)	24(60)	14(37)	**0.04**
rs1801131 (*MTHFR*, methylenetetrahydrofolate reductase)	AA or AC	71(93)	37(95)	34(92)	0.62	AC or CC	48(63)	25(64)	23(62)	1.00
rs1805087 (*MTR*, B12-dependent methionine synthase)	AA or AG	70(96)	35(95)	35(97)	0.56	AG or GG	32(44)	15(41)	17(47)	0.50
rs1801394 (*MTRR*, methionine synthase reductase)	AA or AG	52(68)	28(72)	24(63)	0.39	AG or GG	66(86)	36(92)	30(79)	0.20

N o t e. Data are presented as n (%); the percentage of patients having different allelic variants of the studied SNPs was calculated based on the number of patients for whom each specific allelic variant was successfully determined (accounting for available missing data). SNP — single nucleotide polymorphism.

The allele frequency distribution in the no-reflow group did not deviate from Hardy–Weinberg equilibrium for all studied SNPs (p>0.05). However, in the group of patients without no-reflow, deviations from Hardy– Weinberg equilibrium were noted for SNPs rs1403543 (*AGTR2* gene), rs1799983 (*eNOS*), and rs1801394 (*MTRR*) (p<0.05).

### Creation and evaluation of a genetic prognostic scale

To create a genetic scale predicting the development of no-reflow, SNPs were selected for which differences between the study groups in a univariate analysis were statistically significant (see Table 2). SNPs with more than 5% missing values, for which Hardy– Weinberg equilibrium was not maintained, and for which the proportion of alternative alleles was less than 5% were excluded from the scale. Among the coding variants, preference was given to the recessive genetic model, as this model yielded the maximum number of statistically significant differences between the groups.

As a result, according to the criteria specified above, three SNPs were included in the scale: rs4961 (*ADD1* gene), rs1799998 (*CYP11B2* gene) and rs1801133 (*MTHFR* gene). It should be emphasized that the allelic variants of these SNPs were used, which, within the framework of the recessive model, were associated specifically with an increased risk of developing noreflow: for rs4961 in the *ADD1* gene, genotypes containing an alternative allele (T) — GT or TT; for rs1799998 in the *CYP11B2* gene, a genotype containing only reference alleles (C) — CC; for rs1801133 in the *MTHFR* gene, also a genotype containing only reference alleles (C) — CC. For the above-mentioned genotypes of the selected SNPs, the OR and 95% CI for the development of no-reflow were calculated: rs4961 (*ADD1*), GT or TT genotypes — 2.83 (1.12–7.19), p=0.03; rs1799998 (*CYP11B2*), CC genotype — 5.33 (1.55–18.30), p=0.008; rs1801133 (*MTHFR*), CC genotype — 4.00 (1.13–14.17), p=0.03.

The scale for predicting the development of no-reflow during PCI in patients with STEMI was compiled as follows. A point was assigned if the patient had any of the above-mentioned allelic variants of the SNPs associated with an increased risk of no-reflow. After assessing all three SNPs, the scores were summed. Thus, the maximum score was 3 (associated with the highest risk of developing no-reflow), and the minimum was 0 (associated with the lowest risk of developing no-reflow). Of the 77 patients (taking into account any missing data), the scores in the sample were distributed in the following way: 0 points — 21 patients (27%), 1 point — 20 (26%), 2 points — 25 (33%), and 3 points — 11 (14%). The OR for developing no-reflow for the proposed scale was 2.93 (1.42–6.02), p=0.004. The OR for the simultaneous presence of all three genotypes associated with noreflow was 10.00 (1.28–78.12).

**Figure 1.**
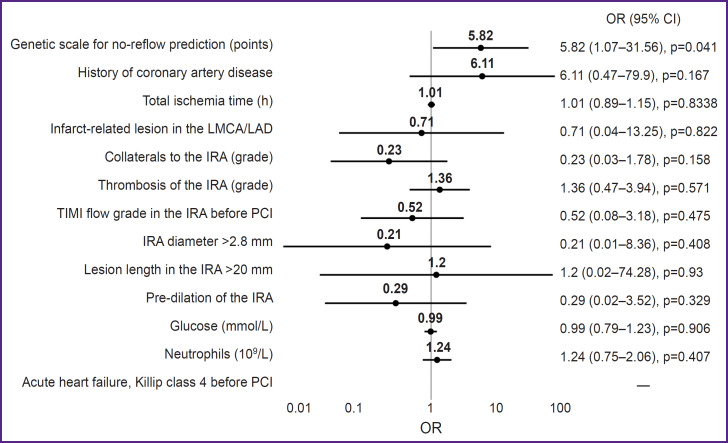
Multivariate analysis of no-reflow predictors incorporating the proposed genetic scale Multivariate analysis confirms the role of the proposed genetic scale as an independent predictor of no-reflow development in myocardial infarction. Other no-reflow predictors were selected for analysis based on various prognostic models [4–9]. CI — confidence interval; IRA — infarct-related artery; LMCA — left main coronary artery; OR — odds ratio; LAD — left anterior descending artery; PCI — percutaneous coronary intervention; TIMI — thrombolysis in myocardial infarction (study group)

The results of the multivariate analysis are presented in Figure 1. Taking into account the influence of confounders, the OR for the development of no-reflow for the proposed model was 5.82 (1.07–31.56), p=0.04.

**Figure 2.**
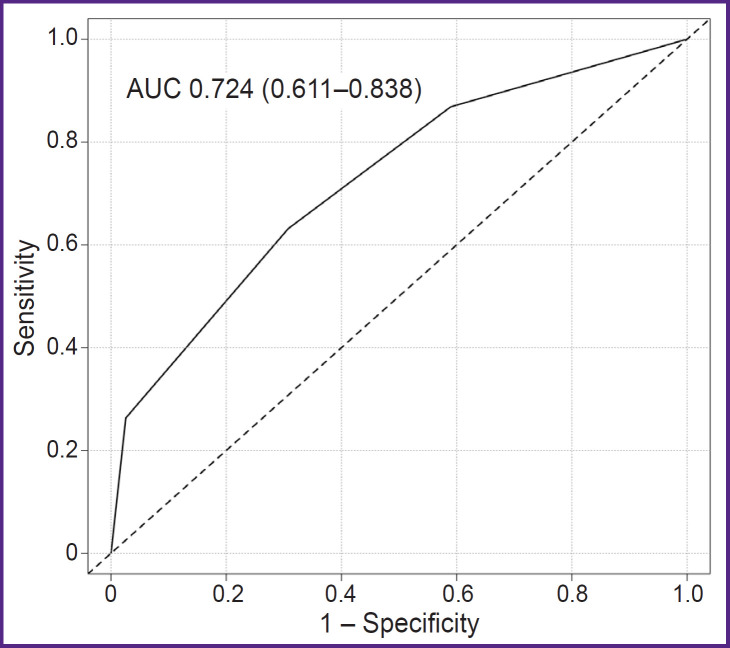
ROC curve of the proposed genetic scale for predicting no-reflow The ROC analysis results indicate a reasonably high predictive value of the developed model. AUC — area under the ROC curve

According to the results of the ROC analysis of the proposed genetic scale, the AUC was 0.724 (0.611– 0.838) (Figure 2). The calculation of the scale’s main prognostic metrics is presented in Table 3.

**T a b l e 3:** Metrics of the proposed genetic scale for predicting no-reflow

Threshold value	Prevalence	True positive	False positive	True negative	False negative	Accuracy	Sensitivity	Specificity	Positive predictive value	Positive predictive value	F-score
3	0.14	10	1	38	28	0.62	0.26	0.97	0.91	0.58	0.41
2	0.47	24	12	27	14	0.66	0.63	0.69	0.67	0.66	0.65
1	0.73	33	23	16	5	0.64	0.87	0.41	0.59	0.76	0.70
0	1.00	38	39	0	0	0.49	1.00	0.00	0.49	—	0.66

A patent was obtained for the created genetic scale [21].

## Discussion

### Interpretation of results

According to the literature [2, 3, 13, 14], the main mechanisms of no-reflow development in MI are ischemic injury (extravasal compression of the microvascular bed), distal microembolization (obstruction of the microvascular lumen from the inside), and endothelial dysfunction (dysregulation of vascular wall tone and permeability). Some of these mechanisms are associated with commonly available parameters routinely determined in real-world clinical practice. Ischemic injury is primarily associated with the timing of reperfusion, lesion location, collateral development, and blood flow in the IRA before PCI. The risk of distal microembolism is largely determined by the volume of thrombotic masses and the PCI tactics. However, there are no parameters clearly associated with the presence and severity of endothelial dysfunction in routine clinical practice. Acute glycemia level depends on many factors and is only indirectly related to endothelial function [2, 3, 14]. There are also no markers characterizing the structure of intracoronary thrombus and the risk of its fragmentation.

For our study, we selected five groups of SNPs, the analysis of which could provide the missing information on the aforementioned mechanisms of no-reflow formation. We analyzed SNPs associated with the renin- angiotensin-aldosterone system, endothelial function, the coagulation cascade, platelet function, and folate metabolism. Statistically significant differences were found for allelic variants of three SNPs from different groups (see Table 2).

The no-reflow group had a statistically significantly higher number of patients with alternative alleles of the rs4961 SNP (GT or TT genotypes, p=0.02). This SNP is localized in the *ADD1* gene, which is responsible for the synthesis of α-adducin. This protein is part of the cytoskeleton, participates in the transport of ions across the cell membrane, and largely ensures the stability of the endothelial barrier [22]. The substitution of the nucleotide guanine (G) for thymine (T) alters the structure of α-adducin (the amino acid glycine is replaced by tryptophan). The association of rs4961 with arterial hypertension has been well studied. The altered protein activates sodium-potassium adenosine triphosphatase in the renal tubules and promotes sodium retention in the organism [23]. An association of rs4961 with no-reflow has not been previously described. We hypothesize that the association of rs4961 with no-reflow can be explained by the influence of this SNP on the development of endothelial dysfunction [22], which is part of the pathogenesis of no-reflow [2, 3, 14].

Statistically significant differences were also obtained when analyzing the SNP rs1799998. The frequency of the CC allelic variant was higher among patients with noreflow (p=0.006). This SNP is localized in the *CYP11B2* gene, which encodes aldosterone synthase, a key enzyme of the renin-angiotensin-aldosterone system that regulates the synthesis of the hormone aldosterone. Data on the effect of the CC genotype rs1799998 on aldosterone synthesis vary. There are studies showing an association between the CC genotype and excessive aldosterone production and, as a consequence, arterial hypertension, decreased excretion of sodium ions, and fluid accumulation in the interstitial space [24]. There are also studies with the opposite result [25]. Information on the association of rs1799998 with the development of no-reflow is not presented in the literature. However, it can be hypothesized that the tendency toward interstitial fluid accumulation, characteristic of the CC allelic variant, plays a role in the development of extravasal compression of the microvascular bed in the no-reflow phenomenon.

The *MTHFR* gene regulates the activity of a key enzyme in the folate cycle, methylenetetrahydrofolate reductase. This enzyme plays a major role in converting folic acid into its bioavailable derivative, 5-methyltetrahydrofolate. Homocysteine metabolism is closely linked to the folate cycle, during which 5-methyltetrahydrofolate is reduced and the methyl group is transferred to vitamin B12 and then to homocysteine, forming the amino acid methionine [26]. The presence of the rs1801133 SNP in the *MTHFR* gene is associated with low methylenetetrahydrofolate reductase activity and correspondingly high serum homocysteine levels, which ultimately leads to endothelial dysfunction, oxidative stress, inflammation, and increased thrombus formation [26]. A link between homocysteine and noreflow has been proven [27]. Moreover, folate cycle dysfunction impairs the methylation of deoxyribonucleic acids, which also contributes to the development of coronary heart disease [26].

Current data on the role of the rs1801133 SNP are contradictory. Some authors point to a link between CT and TT allelic variants and the MI development [26], especially in patients with diabetes and endothelial dysfunction [28]. Other studies demonstrate a link between rs1801133 and no-reflow [29]. However, studies have been published that do not confirm these patterns [30]. Some studies have shown a link between homocysteine levels and perioperative myocardial injury, but no association has been established between injury and the rs1801133 variant [31].

In our study, we obtained a somewhat unexpected result. We also established an association of the rs1801133 SNP with the development of no-reflow, but for the CC genotype (p=0.04). This is at odds with other studies [26, 28, 29], where this genotype is associated with a favorable prognosis. There may be several explanations for this result. All of the above studies [26, 28, 29] were obtained on a European patient population. However, there is evidence that the frequencies of rs1801133 alleles in Russian residents may differ significantly [32]. In addition, the cause-and-effect relationships between the rs1801133 allelic variant and the development of no-reflow are more complex than indicated above [29, 30]. It is known that the process of homocysteine processing is significantly influenced by the levels of folic acid and vitamin B12 consumption by the patient. Absorption of these metabolites may be further limited by chronic *Helicobacter pylori* infection [33]. Studies have shown a direct link between this infection and the development of no-reflow [33]. Chronic inflammation caused by *Helicobacter pylori* predisposes to the development of obstructive and nonobstructive coronary heart disease [34]. Susceptibility to this infection, in turn, may depend on the SNP variant rs1801133 [35].

The conducted multivariate analysis has confirmed that the resulting scale is an independent predictor of noreflow development. All significant predictors of no-reflow development available in our routine clinical practice were selected for analysis. The parameters for the analysis were taken from large prognostic models of recent years [4–9]. Some predictors used in these models were not analyzed because they were not included in our STEMI patient evaluation protocol in the emergency room (activated clotting time, lymphocytes, pre-PCI ejection fraction, creatine phosphokinase, and D-dimer).

In conclusion, based on the obtained scale characteristics (see Table 3), the optimal threshold value for the proposed model should be considered the presence of at least one of the indicated SNPs (the F-score is then maximized at 0.70). If all three SNPs are present in a single patient, the positive predictive value will be maximized at 0.91.

### Limitations and prospects

This study has several limitations. The sample size (80 patients) is relatively small for studies searching for genetic predictors. However, we performed a multivariate analysis that took into account most significant clinical, laboratory, and instrumental predictors of no-reflow development. The analysis confirmed the role of the created scale as an independent predictor of no-reflow.

Based on the literature, it can be assumed that the identified genetic markers lead to the no-reflow development through complex biochemical mechanisms. Although many potential confounders were controlled for in this study, it is clear that not all factors influencing the pathophysiological mechanisms of no-reflow development were considered. Some predictors were not analyzed for organizational reasons; the need to consider others became apparent only after the study was completed.

For a number of SNPs in the control group, Hardy– Weinberg equilibrium was not observed. This is likely due to the fact that the “control” group was artificially matched to the “case” group by gender and age, which likely introduced bias. Furthermore, some samples were damaged during transportation. However, it should be emphasized that the SNPs for which there were concerns were not included in the scale. Also noteworthy is the data on the rs1801133 SNP, which somewhat contradicts previously published studies [26, 28, 29]. Given all of the above, it should be underlined that the obtained results require confirmation in larger studies, and the developed scale requires external validation on an independent sample.

Despite the stated limitations, the study has theoretical and practical value. From a theoretical perspective, the study’s results provide new information on the pathogenesis of no-reflow and expand the range of tools for predicting this complication. The prospects for practical application are varied. For example, implementing the concept of personalized medicine [36] by integrating the proposed genetic model into existing clinical scales will significantly improve the quality of noreflow prediction. Furthermore, in the future, it will be possible to create a genetic test system based on this scale, the use of which directly in the catheterization laboratory will allow for accurate assessment of the risk of no-reflow and modification of surgical tactics to prevent the development of this complication.

## Conclusion

The development of the no-reflow phenomenon during PCI for STEMI is associated with certain allelic variants of three SNPs: GT or TT for SNP rs4961 in the *ADD1* gene, CC for rs1799998 in the *CYP11B2* gene, and CC for rs1801133 in the *MTHFR* gene. These SNPs are associated with various pathophysiological mechanisms of no-reflow development and relate to the renin- angiotensin-aldosterone system (*ADD1*), endothelial function (*CYP11B2*), and the folate cycle (*MTHFR*). The obtained SNPs are combined into the genetic prognostic score (one point for each allelic variant associated with no-reflow). With a maximum sum of three points, the positive predictive value of a result reaches 0.91.
